# Transcriptome Analyses of *Candida albicans* Biofilms, Exposed to Arachidonic Acid and Fluconazole, Indicates Potential Drug Targets

**DOI:** 10.1534/g3.120.401340

**Published:** 2020-07-06

**Authors:** Oluwasegun Kuloyo, Ruan Fourie, Errol Cason, Jacobus Albertyn, Carolina H. Pohl

**Affiliations:** Department of Microbial, Biochemical, and Food Biotechnology, University of the Free State, Bloemfontein, South Africa 9301

**Keywords:** Biofilm, *C. albicans*, arachidonic acid, fluconazole

## Abstract

*Candida albicans* is an opportunistic yeast pathogen within the human microbiota with significant medical importance because of its pathogenic potential. The yeast produces highly resistant biofilms, which are crucial for maintaining infections. Though antifungals are available, their effectiveness is dwindling due to resistance. Alternate options that comprise the combination of existing azoles and polyunsaturated fatty acids, such as arachidonic acid (AA), have been shown to increase azoles susceptibility of *C. albicans* biofilms; however, the mechanisms are still unknown. Therefore, transcriptome analysis was conducted on biofilms exposed to sub-inhibitory concentrations of AA alone, fluconazole alone, and AA combined with fluconazole to understand the possible mechanism involved with the phenomenon. Protein ANalysis THrough Evolutionary Relationships (PANTHER) analysis from the differentially expressed genes revealed that the combination of AA and fluconazole influences biological processes associated with essential processes including methionine synthesis and those involved in ATP generation, such as AMP biosynthesis, fumarate metabolism and fatty acid oxidation. These observations suggests that the interference of AA with these processes may be a possible mechanisms to induce increased antifungal susceptibility.

*Candida albicans* is a leading cause of bloodstream infections in immunocompromised individuals, due to the ability to form biofilms both on living tissues and medically implanted devices (Douglas 2003). The infections caused by *Candida* species are often life-threatening, with a mortality rate between 30 and 50%, with more than 400,000 cases reported yearly (Brown *et al.*, 2012; Mayer *et al.*, 2013). Over the years, several types of antifungals, such as polyenes, pyrimidine analogs, azoles, allylamines, and echinocandins, have been developed to combat the infections (Mathé and Van Dijck 2013). However, from these antifungal classes, the azoles, polyenes, and echinocandins are the most commonly administered with azoles being the predominant choice for treating candidiasis (Denning and Hope 2010; Ostrosky-Zeichner *et al.*, 2010).

The frequent use of azoles in the treatment of candidiasis stems from its excellent bioavailability and reduced toxicity (Meis and Verweij 2001; Denning and Hope 2010; Ben-Ami 2018). Unfortunately, the prominent use has generated a challenge of high antifungal resistance. The known resistance mechanisms employed by *C*. *albicans* biofilm against azoles include the reduction of ergosterol and increased expression of drug efflux pumps (Chandra *et al.*, 2001; Shapiro *et al.*, 2011; Taff *et al.*, 2013). Therefore, the treatment of *C*. *albicans* biofilm infections requires alternative and improved treatment options.

Saturated and unsaturated fatty acids have been documented to have antifungal properties. These compounds can naturally insert themselves into the lipid bi-layer to cause membrane disruption and increase membrane fluidity, which ultimately results in the release of intracellular components (Pohl *et al.*, 2011). The polyunsaturated fatty acids (PUFAs), arachidonic acid (AA) (C20:4 n-6), and eicosapentaenoic acid (EPA) (C20:5 n-3) have been reported to inhibit *C*. *albicans* biofilms (Thibane *et al.*, 2010, 2012). Arachidonic acid and EPA also increases the susceptibility of *C*. *albicans* biofilms to azole antifungals (Ells *et al.*, 2009). However, the molecular mechanism involved remains unknown.

Therefore, this investigation aims to unravel the mechanism involved by conducting transcriptome analysis on *C*. *albicans* biofilms, grown in the presence of AA, fluconazole, or a combination of the two to identify differentially expressed genes under the different conditions.

## Materials and Methods

### Stock solutions

Stock solutions of fluconazole (Sigma-Aldrich, USA) and the fatty acid, AA (Sigma-Aldrich, USA) were dissolved in dimethyl sulfoxide (DMSO) (Sigma-Aldrich, USA) and ethanol (EtOH) (Merck, RSA), respectively. Working concentrations were added to the media, ensuring that the solvent concentration was below 1%. Fluconazole was added to a final concentration of 1 mg.L^-1^_,_ while a concentration of 1 mM was used for AA.

### Yeast strain and cultivation

*Candida albicans* CBS 8758 (SC5314), obtained from the UNESCO MIRCEN culture collection at the University of the Free State was revived on Yeast malt (YM) extract agar (10 g.L^-1^ glucose, 5 g.L^-1^ peptone, 3 g.L^-1^ yeast extract, 3 g.L^-1^ malt extract and 16 g.L^-1^ agar bacteriological) at 30° for 24 h.

### Biofilm formation and storage

The revived *Candida albicans* CBS 8758 (SC5314) was used to inoculate 10 mL Yeast Nitrogen Base (YNB) broth (6.7 g.L^-1^ YNB, 10 g.L^-1^ glucose) and incubated on a shaker at 30° for 24 h. The cells were harvested by centrifugation at 3080 *g* for 5 min and washed thrice with sterile phosphate buffer saline (PBS) (Sigma-Aldrich, USA). Cell count was determined with a hemocytometer, and the cells diluted to 1 × 10^6^ cells.mL^-1^ in 20 mL sterile RPMI 1640 media (2 g.L^-1^ glucose) (Sigma-Aldrich, USA). Biofilm formation was initiated in a polystyrene Petri dish and incubated without agitation at 37° for 90 min, then washed with PBS to remove non-adherent cells (Mishra *et al.*, 2014). The medium was replenished with 20 mL RPMI medium containing the desired concentration of fluconazole with AA, or an equivalent volume of the drug vehicles, which was below 1%. The investigated conditions include **A**A (dissolved in EtOH) and **D**MSO (DA), **f**luconazole (dissolved in DMSO), and **E**tOH (FE) as well as **f**luconazole (dissolved in DMSO) and **A**A (dissolved in EtOH) (FA). The control contained both solvents, **D**MSO, and **E**tOH (DE). A total of 5 biofilms were prepared in triplicate for each condition. The plates were covered with parafilm before incubation for 6 h at 37° to allow for biofilm formation. The biofilms and the growth medium used were scraped off the surface of the plate using a cell scraper and centrifuged at 1971 *g* for 3 min at 4°. The supernatant was aspirated and replaced with 2 mL of Qiagen RNA*later* (Qiagen) to prevent RNA degradation before storage at -80°.

### Total RNA extraction and sequencing

Stored biofilms samples were thawed on ice and centrifuged at 4000 *g* for 5 min. RNA*later* was aspirated, and total RNA extraction was carried out using the RNeasy protect mini kit (Qiagen). At the same time, the DNA present was removed with RNase-Free DNase Set (Qiagen), according to the manufacturer’s instructions. The quality of RNA in each sample was determined at the Centre for Proteomic and Genomic Research (CPGR) before sequencing. Quality tests carried out include checking for contaminants with the NanoDrop ND1000, absolute concentration using the Qubit RNA HS Assay Kit, and an Agilent Bioanalyzer Nano Assay to evaluate the sample integrity. Samples having contaminations were cleaned up using an Agencourt RNAClean XP kit (Beckman Coulter), and the quality confirmed. Ribosomal RNA was removed from 1 µg of RNA per sample, using the Illumina Ribo-zero rRNA removal kit, and purified with an Agencourt RNAClean XP kit. Indexed libraries were prepared using the ScriptSeq v2 RNA-Seq Library Preparation Kit and ScriptSeq Index PCR Primers-Set 1 (Illumina). The library size was profiled with the Bioanalyzer High Sensitivity Assay Kit (Agilent) and quantified with the Qubit HS DNA Assay Kit. The samples were diluted and spiked with a Phix control library (Illumina). Sequencing was completed on a Nextseq 500 (Illumina) using the Nextseq 500 High Output (150 cycles) kit, which yielded paired-end reads with 96% and 97% of total data having bases with a Phred score % ≥ Q30.

### Sequence analysis for differentially regulated genes

Fastq files, obtained from the sequencing, were analyzed for quality using FastQC v0.11.5; (Andrews 2010) and the reads with low quality (<Q30) were discarded, using PRINSEQ-lite v0.20.4 (Schmieder and Edwards 2011). The sequences were aligned to the *C*. *albicans* SC5314 genome assembly 21 (Skrzypek *et al.*, 2017) using TopHat2 (Trapnell *et al.*, 2012; Kim *et al.*, 2013) with the fr-secondstrand, -r 250 mate-std- I 10000 -G option (Dutton *et al.*, 2016). Subsequently, the aligned files were merged with SAMtools (Li *et al.*, 2009). Gene expression count tables were built from BAM files, created with TopHat2 using the BEDTools multicov command (Quinlan and Hall 2010). These counts were used to evaluate the differential expression of genes with DESeq2 (Love *et al.*, 2014). Cuffdiff (Trapnell *et al.*, 2012) was also used to generate differential expression data using aligned reads obtained from TopHat2 (Trapnell *et al.*, 2012; Kim *et al.*, 2013) which were assembled with Cufflinks and merged with Cuffmerge (Trapnell *et al.*, 2012). Differentially expressed genes identified from DESeq2 and Cuffdiff with a *P* ≤ 0.05 were considered significant. Heat maps, volcano plots, and principal component analysis were done as described by Love and co-workers (2014) with a modification of the script as described on github.com/stephenturner. The Protein ANalysis THrough Evolutionary Relationships (PANTHER) classification system was used for enrichment analysis of biological processes of the significant differentially regulated genes in the different data sets (Ashburner *et al.*, 2000; Thomas 2003; Mi *et al.*, 2017; The Gene Ontology Consortium 2019).

### NanoString analysis

RNA extracted from the different treatment conditions [DA= AA (dissolved in EtOH) and DMSO, FE = fluconazole (dissolved in DMSO) and EtOH, FA = fluconazole (dissolved in DMSO), AA (dissolved in EtOH), and DE = the solvents, DMSO and EtOH] were analyzed with the NanoString nCounter analysis system (Geiss *et al.*, 2008) at the University of the Witwatersrand, Department of Internal Medicine, using a gene expression TagSet that targets 36 genes including three housekeeping genes (*orf19.1191*, *SLF1*, *orf19.2184*). Custom-designed TagSets, which comprise a fluorescent-labeled reporter tag and a biotinylated universal capture tag, were designed for each gene. Both the reporter tag and capture tag (referred together to as CodeSet) was hybridized with 100 ng of total RNA at 67° in a preheated thermocycler for 16 h. Subsequently, the hybridized samples were transferred into an nCounter *SPRINT* Cartridge and loaded into a nCounter *SPRINT* Profiler to quantify the transcripts. The nCounter raw expression data file (.RCC) obtained was uploaded into the nSolver Analysis Software 4.0 for review of quality control metrics. The data were normalized by subtracting the geometric mean of the negative control of each sample from the raw counts. The data were grouped between the experiments and control, and their expression ratio was determined. The Log_2_ fold change obtained with nSolver was compared to the values obtained with RNA-seq analysis.

### Data availability

Figure S1 are graphs showing the inhibitory effect of fluconazole, arachidonic acid and the combination of fluconazole and arachidonic acid. Figure S2 is a principle component analyses of the data and Figure S3 is a volcano plot showing the statistical significance *vs.* the magnitude of change between the control sample and the different test conditions. Table S1-S9 comprises the PANTHER classification gene ontology terms for the different test conditions. Table S10 shows a comparison between the transcripts obtained using RNA-Seq and NanoString. All raw sequences and analyzed data for this investigation have been deposited in the NCBI’s GEO database under the accession number GSE137423. Supplemental material available at figshare: https://doi.org/10.25387/g3.12473012.

## Results and Discussion

### Different conditions produced unique transcriptome profiles

RNA sequencing has become an important technique to understand genomic functions (Hrdlickova *et al.*, 2017). The most common application of RNA-seq is for the identification of differentially regulated genes under two or more conditions (McDermaid *et al.*, 2018). In this study, RNA-seq was applied to identify differentially regulated genes in *C*. *albicans* biofilms grown in fluconazole, AA, or the combination of the two, compared to the drug vehicles as controls after early biofilm formation (6 h). The use of 1 mM AA was based on our observation that AA concentrations below 1 mM were non-inhibitory to *C. albicans* biofilms (Figure S1a) as well as the report by Ells and co-workers (2009), which reported an increase in susceptibility of *C*. *albicans* biofilms to 1.25 mg.L^-1^ clotrimazole increased by approximately 160% in the presence of 1 mM AA, in comparison with biofilms without AA. We made a similar observation with fluconazole in combination with 1 mM AA after measurement of biofilm metabolic activity using XTT assay (Figure S1b). The most significant (*P* ≤ 0.05) increase in susceptibility was observed when 1 mM AA was combined with 1 mg.L^-1^ of fluconazole assay (Figure S1c). Mishra and co-workers (2014) demonstrated that a combination of 500 mM AA and half the fluconazole MIC did not cause a significant increase in fluconazole susceptibility, indicating that the presence of high AA concentration is vital to the observed increase in fluconazole susceptibility. Although the AA concentration used is much higher than the physiological concentration, which is between 0.1-50 µM in human plasma (Pompeia *et al.*, 2003; Tallima and Ridi 2018), the aim of this study was to investigate the antifungal mechanism of AA, thus the higher, inhibitory concentration was used.

The independent Illumina sequencing runs of *C*. *albicans* biofilms biological replicates produced a total of 325 million raw reads, with 92.4% having a Phred score % ≥ Q30. Principal component analysis between the different replicates obtained showed a close correlation between the number of reads obtained for each run, as shown by the close clustering of the spots, which represents individual runs (Figure S2). The variation between the different test conditions (FA, DA, and FE) and the control (DE) was minimal, as represented by the distance between the spots. The high reproducibility among the data sets was demonstrated with a heat map that showed close clustering between identical replicates, which can be seen with the corresponding color intensity. The relatively higher variability among the replicates of DE, may be due to higher morphological diversity in these biofilms (*i.e.*, yeast, hyphae) compared to biofilms grown in the presence of AA (Clément *et al.*, 2007) or fluconazole (Ha & White 1999). Volcano plots to show the number of statistically significant differentially regulated genes are shown in Figure S3. As shown, there were more differentially regulated genes in FA and FE than in DA.

The data obtained after RNA-Seq were initially analyzed with Cuffdiff to identify differentially expressed genes from different conditions. The biofilms which were grown in the presence of DE served as the control and was compared with the other three experimental conditions of DA, FA, and FE to identify differentially expressed genes. The open reading frames were identified with pipelines by aligning the reads to the *C*. *albicans* genome haploid assembly 21 from the *Candida* genome database (CGD) (Skrzypek *et al.*, 2017). Generally, Cuffdiff converts aligned read counts to FPKM (Fragments Per Kilobase of exon per Million fragments mapped), then calculates the observed change in expression and the statistical significance. However, the program assumes that the number of reads produced by each transcript is proportional to its abundance (Trapnell *et al.*, 2012). An observation made using Cuffdiff was genes that are close to a particular locus were assigned the same fold change value. Therefore, to resolve this challenge, the data were analyzed with DESeq2, which uses the count matrix (Love *et al.*, 2014).

According to the analysis, the number of significantly regulated (*P* ≤ 0.05) genes in each of the tested conditions differed between Cuffdiff and DESeq2, with DESeq2 identifying more differentially regulated genes than Cuffdiff. Studies to compare the different software packages for differential expression analysis have also reported the presence of more genes using DESeq2 (Nookaew *et al.*, 2012; Seyednasrollah *et al.*, 2015). An evaluation of the performance of different software packages for differential expression analysis conducted by Zhang and co-workers (2014) showed that the performance of DESeq and Cuffdiff2 was similar in the analysis of sequencing dept ≥ 20 million reads. The investigation suggested that if the number of false positives is a concern, differentially regulated genes shared between the two programs should preferably be used for further analysis. Therefore, in this study, only the significantly differentially regulated genes (*P* ≤ 0.05), which intersect between Cuffdiff and DESeq2, were considered for further investigation. In the different conditions tested, the number of genes intersecting between the two programs are indicated in Table 1. Genes with a Log_2_ fold change of more than one (Log_2_ ≥ 1) were identified from the gene list in each of the experimental conditions and for further analysis.

**Table 1:** The number of significant differentially expressed genes identified from different conditions. DA (DMSO and Arachidonic acid), FA (Fluconazole and Arachidonic acid), and FE (Fluconazole and EtOH)

Conditions	Significant regulation		Genes common to Cuffdiff and DESeq2	Genes with fold change (Log_2_ ≥ 1)	
	Cuffdiff	DESeq2		Downregulated	Upregulated
DA	1200	2124	783	183	244
FA	1790	2853	1253	343	407
FE	1089	1954	803	229	312
Total	4079	6931			

**Figure 1:**
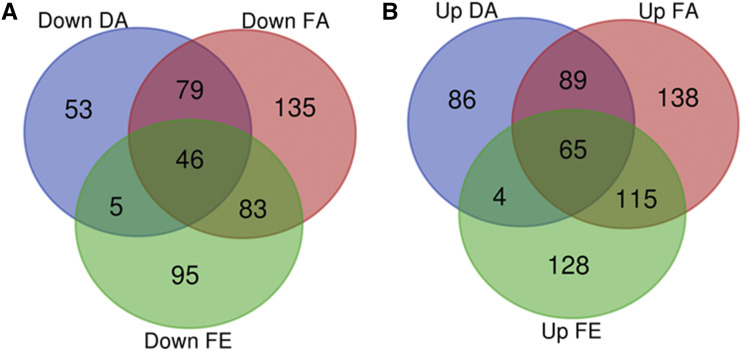
Relationship between differentially regulated genes with a fold change greater than one (Log_2_ ≥ 1) in all tested conditions. (A) Downregulated genes (B) Upregulated genes. DA (DMSO and Arachidonic acid), FA (Fluconazole and Arachidonic acid) and FE (Fluconazole and EtOH).

The distribution of the differentially regulated genes between the different test conditions was investigated (Figure 1). Most genes present are unique to a treatment condition, suggesting that the different treatment conditions may have induced unique processes or proteins.

### Enrichment analysis of differentially regulated genes

**Figure 2:**
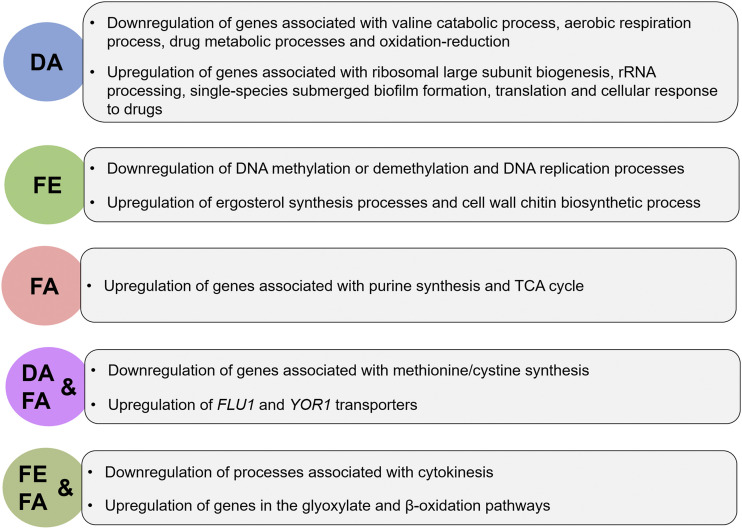
A summary of ontology term processes under each test condition. DA (DMSO and Arachidonic acid), FA (Fluconazole and Arachidonic acid), and FE (Fluconazole and EtOH).

Biological process enrichment analysis was conducted to assign gene ontology (GO) terms to the genes obtained from different conditions, using Protein ANalysis THrough Evolutionary Relationships (PANTHER) Gene Ontology (GO) (Ashburner *et al.*, 2000; Mi *et al.*, 2017; The Gene Ontology Consortium 2019). Different clusters shown on the Venn diagram for both upregulated and downregulated genes were individually analyzed. The downregulated and upregulated genes shared by all the tested conditions were not enriched for specific GO terms, possibly because most of the genes present in this section are yet to be characterized. However, a search of the possible role of the downregulated genes indicates that they are associated with filamentous growth, cell wall, and membrane transport processes, with the upregulated genes linked to roles in transcriptional regulation, secretory functions, and transport. Figure 2 is a summary of processes with enriched GO terms for the different test conditions.

### Processes enriched by growth with arachidonic acid

The unique downregulated genes associated with biofilms grown in the presence of AA (DA) were enriched for four GO terms, namely, valine catabolic process, aerobic respiration process, drug metabolic processes, and oxidation-reduction (Table S1). In the valine catabolic process ontology, *HPD1* (which encodes hydroxypropionate dehydrogenase) and *ALD6* (which encodes malonate aldehyde dehydrogenase) were identified. In *C*. *albicans*, these genes are involved in the degradation of propionyl-CoA via a modified *β*-oxidation pathway to produce acetyl-CoA (Otzen *et al.*, 2014). Propionyl-CoA is a toxic intermediate produced by yeast cells from the breakdown of cholesterol, valine, odd-chain fatty acids, methionine, isoleucine, and threonine (Brock and Buckel 2004; Zhang *et al.*, 2004). Although *C*. *albicans* can use propionyl-CoA as a sole carbon source, glucose is still the preferred carbon source for *C*. *albicans* (Otzen *et al.*, 2014). Therefore, the downregulation of propionyl-CoA degrading genes in biofilms grown in the presence of AA could imply either the sufficient availability of glucose or the absence of propionyl-CoA.

Aerobic respiration GO term was associated with *NDH51* (NADH dehydrogenase complex I (CI)) and *ACO1* (aconitase) genes. NADH dehydrogenase complex I is a primary component of the *C. albicans* mitochondrial electron transport chain (Nosek and Fukuhara 1994). Aconitase, an iron-sulfur containing protein, catalyzes the conversion of citrate to isocitrate in the tricarboxylic acid cycle (Gangloff *et al.*, 1990). The downregulation of these genes could be due to glucose availability because *ACO1* is regulated by catabolite repression in the presence of glucose and glutamate (Gangloff *et al.*, 1990; Flores *et al.*, 2000). Glucose availability also influences *NDH51* expression (McDonough *et al.*, 2002; Vellucci *et al.*, 2007).

The *CAT1* gene, which encodes the catalase enzyme (Wysong *et al.*, 1998), was assigned to the drug metabolic process GO term. Glucose availability has been reported to repress the expression of catalase in *C*. *albicans*, even when treated with hydrogen peroxide (Nakagawa *et al.*, 1999). The downregulation of the genes associated with these processes suggests that the presence of AA may be interfering with glucose uptake and metabolism.

The enriched upregulated genes, which were unique to biofilms grown in the presence of AA, were associated with four GO terms, namely, ribosomal large subunit biogenesis, rRNA processing, single-species submerged biofilm formation, translation and cellular response to drugs (Table S2). The process of ribosomal biogenesis entails highly coordinated procedures with intense energy demands for the synthesis, processing, transport and ribosome assembly (Thomson *et al.*, 2013). DEAD-box proteins *DRS1*, *DBP3*, and *DBP7* are involved in RNA metabolism processes, which include the unwinding of double-stranded RNA molecules (Linder 2006). However, *DRS1*, *DBP3*, and *DBP7* are involved in the maturation of ribosomal RNA (Issi *et al.*, 2017). *Candida albicans* ribosome biogenesis processes are downregulated due to the high energy required when the cell undergoes stress due to reactive oxygen species (ROS) and nutrient depletion (Loar *et al.*, 2004; Kos-Braun and Koš 2017). However, the upregulation of genes associated with ribosomal biogenesis indicates the presence of acceptable energy levels in the cells when grown in the presence of DA. Also, hyphae inducers such as serum and *N*-acetyl-D-glucosamine causes a reduction in ribosomal RNA (Fleischmann and Rocha 2015) and the fact that AA inhibits yeast to hyphae transition in *C. albicans* (Clément *et al.*, 2007; Shareck and Belhumeur 2011), may explain the opposite effect observed here.

Genes that play essential roles in biofilm formation in *C. albicans* such as *CSH1*, *GCA1*, *NDT80*, *ADH1*, *TEC1*, and *TYE7* were assigned to the single-species submerged biofilm formation GO term, which was enriched in the presence of AA. The *CSH1* gene product (Csh1), is a vital virulence factor in *C. albicans*, which affects cell surface hydrophobicity (Antley and Hazen 1988). It is also involved in the irreversible adhesion of cells during the early biofilm formation (Chandra *et al.*, 2001; Bujdáková *et al.*, 2013). Gca1 is a plasma membrane-associated glucoamylase enzyme with a role in the formation of *C. albicans* biofilm matrix (Nobile *et al.*, 2009) and has been reported to assist with starch degradation and energy generation (Maicas *et al.*, 2016). Alcohol dehydrogenase encoding *ADH1* catalyzes the reversible conversion of aldehyde to ethanol (Bertram *et al.*, 1996), and is downregulated in *C. albicans* biofilms (Mukherjee *et al.*, 2006). Transcriptional regulators *NDT80*, *TEC1* and *TYE7* were also associated with this ontology, while *NDT80* and *TEC1* are among the six *C. albicans* biofilm master circuit transcriptional regulators (Nobile *et al.*, 2012), *TYE7* is involved in the activation of the glycolytic pathway (Askew *et al.*, 2009). Transcriptional factors *NDT80* and *TEC1* are required for hyphal growth, although *NDT80* is not always required in all growth conditions, such as during the use of glycerol as a carbon source (Daniels *et al.*, 2015; Min *et al.*, 2018). The upregulation of genes associated with the biofilm formation ontology may be a possible attempt by the yeast to compensate for decreased biofilm formation after treatment with DA (Figure S1a).

### Processes enriched by growth with fluconazole

Gene ontologies enriched in downregulated genes that are unique to biofilms grown in the presence of fluconazole (FE) include DNA methylation or demethylation, negative regulation of helicase activity, DNA replication checkpoint, DNA strand elongation involved in DNA replication and DNA-dependent DNA replication maintenance of fidelity (Table S3). DNA methylation and demethylation processes are epigenetic modification, which represses transcription and transposon control (Finnegan *et al.*, 2000; Martienssen and Colot 2001). In *C*. *albicans*, this process is associated with the repression of genes involved with phenotypic switching, structural genes and gene regulation. Additionally, the presence of environmental stressors can also induce *C*. *albicans* DNA methylation (Mishra *et al.*, 2011). The presence of fluconazole has been associated with stresses that have been explicitly identified as the primary cause for the formation of trimera, leading to aneuploidy in *C*. *albicans* (Harrison *et al.*, 2014). The histone H3 isoform encoding gene, *HHT21*, was associated with the DNA methylation or demethylation ontology. According to an investigation by Zacchi and co-workers (2010), transcription and chromosome segregations are affected by a change in the number of *C*. *albicans* histones, and this results in growth defects and aneuploid formation.

Upregulated genes unique to biofilms grown in the presence of FE were enriched for GO terms, ergosterol biosynthetic process, farnesyl diphosphate metabolic process, and cell wall chitin biosynthetic process (Table S4). Ergosterol is essential in yeast because it assists with the maintenance of membrane fluidity, permeability, and integrity (Lv *et al.*, 2016). Fluconazole inhibits ergosterol synthesis by binding to lanosterol demethylase enzyme, encoded by *ERG11* (Bondaryk *et al.*, 2013). The upregulation of ergosterol synthesis genes is one of the known resistance mechanism of *C. albicans* biofilms against fluconazole treatment (Sanglard *et al.*, 2003; Tsui *et al.*, 2016). Several genes associated with ergosterol synthesis, including *ERG3*, *ERG1*, *ERG11*, *ERG9*, and *CYB5*, were linked with this ontology term. The upregulation of processes associated with ergosterol synthesis in the presence of fluconazole indicates a resistance response to compensate for the presence of the antifungal (Bondaryk *et al.*, 2013).

Cell wall chitin biosynthetic process was also associated with genes upregulated in biofilms grown in the presence of fluconazole. Fluconazole induces membrane and cell wall stress, by sterol disruption and increasing the fluidity of the plasma membrane (Abe *et al.*, 2009; Klis and Brul 2015). In response to cell wall stress, *C. albicans* increases the amount of chitin and expression of cell wall repair proteins (Munro *et al.*, 2007; Sorgo *et al.*, 2011).

### Processes enriched by growth with both fluconazole and arachidonic acid

The downregulated genes, which were unique to biofilms grown in the presence of both fluconazole and AA (FA), were not enriched for any GO term. Most of the genes in this cluster are uncharacterized. However, those with known functions have roles in zinc homeostasis, oxidative stress, and copper and zinc transport.

Upregulated genes, unique to cells grown in the presence FA, are enriched for AMP biosynthetic processes, fumarate metabolic process, ATP synthesis coupled proton transport, fatty acid oxidation, tricarboxylic acid process and acyl-CoA metabolic process (Table S5). Purine synthesis in *C. albicans* occurs via the *de novo* biosynthesis pathway and the salvage pathways (Rolfes 2006). Adenylosuccinate synthase (encoded by *ADE12*), which catalyzes the conversion of inosine monophosphate (IMP) to adenylosuccinate, and adenosine kinase (encoded by *ADO1*), which recycles adenosine to AMP, were assigned to the AMP biosynthetic process ontology. The energy demand for purine biosynthesis is high; therefore, the regulation of this process is via feedback inhibition by the ATP and ADP end products and the presence of excess purine in the cell-extracellular environment (Rébora *et al.*, 2001; Rolfes 2006). The upregulation of genes associated with purine synthesis in biofilms grown in the presence of fluconazole and AA indicates an attempt to increase ATP generation under these conditions.

ATP synthesis coupled proton transport GO term was represented by ATP synthase alpha subunit (encoded by *ATP1*), F1F0-ATP synthase beta subunit (encoded by *ATP2*), oligomycin-sensitive conferring protein (OSCP) (encoded by *ATP5*) and F1F0 ATP synthase subunit h (encoded by *ATP14*), these proteins constitute different components of the mitochondrial ATP synthase, required for the phosphorylation of ADP to ATP using the energy provided by the electrochemical gradient (Arnold *et al.*, 1998; Xu *et al.*, 2015). The absence of *ATP1* or *ATP2* in *C*. *albicans* result in the cells being avirulent, unable to grow on non-fermentable or non-glucose carbon sources, defective in filamentation, biofilm formation, and ATP production (Li *et al.*, 2017; Li *et al.*, 2018). Lack of growth in media containing glycerol and lactate as a carbon source was observed in *Saccharomyces cerevisiae ATP14* homozygous mutant (Arselin *et al.*, 1996). This also supports the possible need for ATP generation in biofilms grown in the presence of both fluconazole and AA.

Fumarate metabolic process and tricarboxylic acid process are represented by genes encoding enzymes within the tricarboxylic acid (TCA) cycle. Fumarate hydratase (encoded by *FUM12*) was assigned to both ontologies. In contrast, adenylosuccinate synthase (encoded by *ADE12*) was assigned to the fumarate metabolic process and malate dehydrogenase (encoded by *MDH1-1*) and succinate-CoA ligase (encoded by *LSC1*) to the tricarboxylic acid process. Fumarase is present in the cytoplasm and mitochondria of eukaryotic cells. The mitochondrial fumarase catalyzes the reversible hydration of fumarate to L-malate in the TCA cycle, while the cytosolic fumarase is synthesized in response to DNA damage (Yogev *et al.*, 2010). The upregulation of genes associated with the TCA cycle in the presence of both fluconazole and AA may also be as a result of the need for an increase in ATP generation.

Putative pyruvate dehydrogenase (encoded by *PDB1*), carnitine acetyltransferase (encoded by *CAT2*), and succinate-CoA ligase (encoded by *LSC1*) were associated with the GO term acyl-CoA metabolic process. The functions of *CAT2* and *LSC1* in the cell have been discussed earlier, but *PDB1*, which encodes a β–subunit of the pyruvate dehydrogenase complex, catalyzes the conversion of pyruvate to acetyl-CoA and CO_2_ (Vellucci *et al.*, 2007). The GO terms enriched in the upregulated genes of *C*. *albicans* cells grown in the presence of fluconazole and AA are mostly associated with energy generation. This suggests that under this treatment condition, the cell attempts to increase its energy output, and this indicates that there is a demand for energy.

### Overlapping processes enriched by growth with either arachidonic acid or fluconazole

The upregulated and downregulated genes shared between biofilms grown in the presence of either AA (DA) or fluconazole (FE) were not enriched for any specific GO terms. The reported descriptions for the downregulated genes include phosphatase complex subunit, SIN3-binding protein, a heme oxygenase, and a high-affinity iron permease, while the upregulated genes are associated with the cell wall and DNA synthesis.

### Overlapping processes enriched by growth with either arachidonic acid or a combination of fluconazole and arachidonic acid

The downregulated genes which were shared between biofilms grown in the presence of either AA (DA) or a combination of fluconazole and AA (FA) were enriched for GO terms, which include hydrogen sulfide biosynthetic process, sulfate assimilation and sulfur amino acid biosynthetic process (Table S6). The different processes mentioned above are represented by ATP sulfurylase (encoded by *MET3*), putative adenylylsulfate kinase (encoded by *MET14*), putative sulfite reductase beta subunit (encoded by *ECM17*) and O-acetylhomoserine O-acetylserine sulfhydrylase (encoded by *MET15*) which are genes in the *C*. *albicans* methionine/cysteine biosynthesis pathway. Methionine/cysteine synthesis is essential for biofilm formation in *C*. *albicans* (García-Sánchez *et al.*, 2004; Murillo *et al.*, 2005). A failure in the formation of proper biofilm, diminished adhesion and a decrease in filamentation was observed in homozygous *ecm17∆/∆ C*. *albicans* strains and with the absence of methionine or cysteine (Li *et al.*, 2013). *MET3*, which is an activator of the methionine/cysteine pathway for sulfur assimilation, is one of the most highly upregulated genes during biofilm formation in *C*. *albicans* (Murillo *et al.*, 2005). However, the presence of methionine or cysteine at a concentration higher than 1 mM represses the expression of *MET3* (Care *et al.*, 1999). The concentrations of methionine and cysteine in RPMI media are 0.43 mM and 0.062 mM, respectively. Hence, the downregulation in methionine/cystine processes may not be due to catabolite repression but rather the presence of AA in both these conditions, which may repress the methionine/cysteine pathway, resulting in low biofilm formation.

The GO terms enriched in upregulated genes shared between biofilms grown in the presence of either DA or FA include xenobiotic transport, spermine transport, and spermidine transport (Table S7). These GO terms were represented by an ATP-binding cassette (ABC) transporter (encoded by *YOR1*) as well as *FLU1*, which encodes for fluconazole resistance 1 protein, an efflux pump belonging to the major facilitator superfamily (MFS) class of transporters involved with drug efflux (Cannon *et al.*, 2009). However, contrary to expectations of its role in fluconazole efflux, the disruption of *FLU1* in *C. albicans* has a negligible effect on its fluconazole susceptibility, although its deletion causes susceptibility to mycophenolic acid (Calabrese *et al.*, 2000).

The *C. albicans* Yor1 is similar to the *S. cerevisiae* Yor1, which is an ATP-binding cassette (ABC) transporter, which acts as an oligomycin resistance permease (Gaur *et al.*, 2005). However, Yor1 in *C*. *albicans* effluxes beauvericin, a compound that enhances azole susceptibility, from the cell (Shekhar-Guturja *et al.* 2016). Although the upregulated genes associated with this GO term in DA and FA are involved in transport, their role in the transport of either fluconazole or AA has not been identified.

### Overlapping processes enriched by growth with either fluconazole or a combination of fluconazole and arachidonic acid

The GO terms enriched for downregulated genes shared between biofilms grown in the presence of either fluconazole (FE) and a combination of fluconazole and AA (FA) include filamentous growth regulation and cytoskeleton-dependent cytokinesis (Table S8). The genes linked with the filamentous growth ontology include Zn(II)2Cys6 transcription factors (encoded by *ROB1*, *AHR1*, and *UME6*) as well as a G-protein alpha subunit (encoded by *GPA2*). Biofilm formation in *C. albicans* is under the control of six master circuit transcriptional factors, including Rob1 (Nobile *et al.*, 2012). The importance of *ROB1* to *C. albicans* biofilm formation is demonstrated with the failure of *rob1∆/∆* mutants to form biofilms and the haploinsufficiency of the heterozygous mutant (Nobile *et al.*, 2012; Glazier *et al.*, 2017). The zinc cluster transcription factor, Ahr1, activates adhesion, and hyphal regulatory genes (Askew *et al.*, 2011). Cell adhesion to host surface and hyphae formation are essential processes of *C. albicans* biofilms formation and also contributes to the biofilm structure (Chandra *et al.*, 2001; Finkel and Mitchell 2011). *UME6* is an essential regulator for hyphal extension and virulence in *C. albicans* (Banerjee *et al.*, 2008). This is also seen in the enrichment of the biological adhesion GO term, which was represented by agglutinin-like protein 3 (encoded by *ALS3*), Zn(II)2Cys6 transcription factor (encoded by *UME6*), and the zinc cluster transcription factor (encoded by *AHR1*). *Candida albicans* agglutinin-like protein 3 (Als3) is known to function in adhesion, biofilm formation, and iron acquisition processes (Almeida *et al.*, 2008; Liu and Filler 2011). However, its expression is only observed in the hyphae and pseudohyphae (Hoyer *et al.*, 1998). The deletion of *ALS3* in *C. albicans* resulted in the formation of thin and disorganized biofilms *in vitro* (Nobile *et al.*, 2006). The downregulation of critical transcriptional regulatory genes in biofilms grown in FE and FA may contribute to the presence of poorly formed biofilms under these conditions (Figure S1b).

The cytoskeleton-dependent cytokinesis GO term was represented by septin (encoded by *CDC10*), formin-binding protein (encoded by *HOF1*), and Ras GTPase-activating protein (encoded by *IQG1*). Septins are filament-forming proteins which comprise Cdc3, Cdc11, Cdc12 and Cdc10. (Longtine *et al.*, 1996; Frazier *et al.*, 1998). Although *C*. *albicans* viability or growth rate is not affected by a mutation of *CDC10*, the observed defects in *C*. *albicans CDC10* mutants include a failed cytokinesis due to abnormal cell morphology of elongation and enlargement of cells without separation as well as defects in chitin localization (Warenda and Konopka 2002). *HOF1* encodes an F-box protein involved in cytokinesis and the formation of a ring around the bud neck of the mother and daughter cells. In the absence of *HOF1*, *C. albicans* cells suffer severe cytokinesis and separation defects, which is evident in the formation of chains of cells (Li *et al.*, 2008; Jonkers and Rep 2009). *IQG1* also localizes to the bud neck, contributing to neck formation with the assembly and contraction of the actomyosin ring. In the absence of *IQG1*, cell necks are swollen and broaden (Li *et al.*, 2008). The downregulation of processes associated with cytokinesis process in biofilms grown in the presence of fluconazole as well as a combination of fluconazole and AA may be due to the presence of fluconazole as it has been shown to induce trimeras as discussed previously (Harrison *et al.*, 2014).

The GO term enriched in the upregulated genes shared by biofilms grown in FE) and FA includes the glyoxylate cycle, acetate catabolic process, carbon utilization and fatty acid catabolic process (Table S9). All these processes were represented by the isocitrate lyase (encoded by *ICL1*), malate synthase (encoded by *MLS1*), and a multifunctional enzyme of the β-oxidation pathway (encoded by *FOX2*). *ICL1* and *MLS1* are the first two genes of the glyoxylate cycle, an alternative pathway that allows the conversion of two-carbon acetate into a four-carbon dicarboxylic acid, which replenishes the TCA cycle or serves as a precursor for either amino acid or carbohydrate synthesis (Kunze *et al.*, 2006).

Conversely, Fox2 catalyzes both enoyl-CoA hydratase and 3-hydroxyacyl-CoA dehydrogenase activities in the β-oxidation pathway (Hiltunen *et al.*, 2003). Alternative energy-generating pathways, such as the β-oxidation pathway and the glyoxylate pathway, allow the yeast to grow on alternative carbon sources such as fatty acids, ethanol and acetate (Kunze and Hartig 2013). Treatment of *C*. *albicans* cells with fluconazole has been shown to increase metabolites connected with the central carbon metabolic pathway (Katragkou *et al.*, 2016). Therefore, the upregulation of glyoxylate pathway genes and *FOX2* from the β-oxidation pathway may indicate an increase in the energy requirements of the biofilms under these conditions.

### Confirmation of RNA-Seq data with NanoString

The fold change obtained using RNA-Seq compared with that from NanoString, using selected genes of interest is shown in Table S10. As observed, the similarity between the transcript levels obtained from the two platforms indicates consistency in the data generated for the analysis.

## Conclusions

Differentially regulated genes were obtained in all the tested conditions with the most differentially regulated genes obtained with the combination of fluconazole and AA (FA). Analysis of the biological processes indicated that in the presence of AA (DA), energy generation processes were downregulated, which may be due to an inhibition of glucose uptake. The low energy generation in the presence of AA may also be associated with the possible upregulation of biofilm formation processes to compensate for low biomass. As expected, the presence of fluconazole enriched for processes involved in ergosterol synthesis and an increase in chitin content. Also, processes associated with abnormal cell cycle, such as aneuploidy, was enriched in the fluconazole treated cells. In cells grown with the combination of fluconazole and AA, several processes associated with ATP synthesis was enriched, indicating the need to produce ATP via alternative processes. Methionine/cysteine synthesis processes, which are required for biofilm formation, were downregulated in the presence of AA. It can be suggested that the presence of AA interferes with the energy generation and methionine synthesis processes as a mechanism for increased susceptibility. This lays the ground for more detailed studies regarding these processes as possible drug targets.
